# American Surgical Association

**Published:** 1883-08

**Authors:** E. S. McKee

**Affiliations:** Cincinnati


### Society Reports.

### ARTICLE VI.

AMERICAN SURGICAL ASSOCIATION.

MR. EDITOR:—The fourth annual session of the American Surgical Association convened in Cincinnati, Thursday, May 31st, ult., with S. D. Gross, M.D., LL.D., D.C.L., Oxon, LL.D., Cantab (emeritus) in the chair, and a few number of members present. The president delivered the following address:

FELLOW-MEMBERS OF THE AMERICAN SURGICAL ASSOCIATION: Allow me, as your presiding officer, to congratulate you upon your safe arrival, and to extend to you a cordial welcome to the Queen City, the center of medical education in the Buckeye State, the residence of eminent medical teachers and practitioners, and repository of the ashes of great medical men, who, during their laborious lives, added luster to the profession and left behind them undying names. We have come here not as pleasure-seekers, but as a great body of men bent on earnest work in the interests of our great and noble calling. It is a source of rejoicing to me, as I am sure it is to you, that since our last meeting our ranks have not been invaded by the fell destroyer. On that occasion it was my meloncholy duty to announce to you the demise of some of our fellows, all men of distinction and great usefulness. The meeting upon which we are about to enter cannot fail, if we may judge from our programme, to be of unusual interest and that our deliberations will be conducted with that celerity and harmony of feeling which has hitherto characterized it, I am safe in predicting. Allow me in concluding this brief salutatory, to direct your attention to a proposed alteration of the constitution, to increase the number of fellows from 100 to

150. The change is an important one and merits your serious consideration. I also respectfully suggest that the acting council be increased from four to seven, so as to impart greater efficiency to its work. Finally, it appears to me wise to take action upon any proposed amendments to the constitution and by-laws, on the last day of every session, instead of letting them lie over for one year as now provided for.

Minutes were read and adopted.

In the absence of Prof. E. Beverly, San Francisco, Cal. and Prof. H. F. Campbell, of University of Virginia, members of council, the chair appointed Prof. E. M. Moore, of Rochester, N. Y.

Treasurer reported $2,070.21 on hand.

On motion, council organized to act on applications for membership.

Prof. Gross being both President and first on the programme to read, gave way to the following paper:

I. Have we any Therapeutic Means as Proven by Experiment, which Directly Affect the Local Processes of Inflammation?

By C. B. Nancrede, M. D. Philadelphia. He spoke of blood-letting, more particularly of local blood-letting, the reckless abandonment of which he deplored.

Prof. Campbell, of Augusta, Ga., spoke of it as the lost art; said if it were not for blood, we would have no swelling. Recommended his students to use the lancet much more than formerly. In puerperal convulsions, morphine first then the lancet. He had not bled one-half so often as he should have done.

Dr. Moore said: Paper has much merit, especially as to its scientific exposition of the *modus operandi* of blood-letting.

Dr. Busch who had made his fame from the frequent and successful use of the lancet, fell from its use in pneumonia. In an epidemic of pneumonia where he had practiced, the lancet was used in every case and every patient died; changed to whisky, beef tea and quinine and almost the exact reverse was the case. With perfect diagnosis and absence of anything like typhoid symptoms, the lancet should be used more frequently.

Dr. Post, N. Y.: Can not attach too much importance to gravity in inflammation. Blood-letting should be repudiated in asthenic troubles. In persons of good habits and traumatic conditions, blood-letting is of much use.

Dr. Kinlon, of Charleston, S. C.: Blood-letting has always disappointed me in inflammatory affections and I raise my voice against it.

Prof. Gunn, of Chicago, said: "There are inflammations, and there are inflammations." Must be careful as to diagnosis. In some cases blood-letting is beneficial, others not.

Prof. Brakes, Nashville, Tenn.: In many cases general blood-letting is successful in local disorders and vice versa.

Prof. Gregory, St. Louis: Blood-letting locally is rational in local inflammation. Cannot tell just what vein to open. Is not useful in idiopathic inflammation, but is an injury. We should meet and combat the cause of inflammation. I am one of the old doctors, who come from the time of bleeding. Have seen epidemics of pneumonia where many have died under the lancet, have seen those who have died without the lancet.

Prof. Dawson, Cincinnati: If it be so great a remedy, why have we abandoned it? Does any one here bleed a patient a year? Why advocate what we do not practice? Drugs will accomplish the same thing.

Dr. Nancrede: I have not been thoroughly understood. Have said diagnosis must be accurate, and advised bleeding shortly after or while stasis had taken place. The instant there is tissue change then it is too late. I spoke of surgical inflammation, did not speak of pneumonia etc. I did not recommend blood-letting when heart is feeble. The loss of a night's sleep, a slight diarrhœa, reduces blood-cells one-twentieth. I have no doubt mine are reduced as much from reading this paper. It requires only a few hours to replace them.

II. Lister's System of Antiseptic Wound-treatment, *versus* its Modifications. By B. A. Watson, Jersey City, N. J.

This paper took decided grounds in favor of Listerism.

A number of fellows spoke against Listerism and said that it was not followed by a majority of the practitioners in their cities.

Dr. Nancrede proposed to follow the essentials of Listerism some time yet.

Dr. Prince, Jacksonville, Ills., spoke in favor of Listerism.

Dr. McGraw, Detroit, reported a case which he had lost by carbolic acid poisoning.

Dr. Dawson, Cincinnati: The great thing that Lister has done is to teach us cleanliness. To Dr. T. M. Markoe belongs great credit for giving us thorough drainage.

III. Re-amputation at the Hip Joint; Secondary Hæmorrhage; Ligation of the Primitive Iliac Artery; Remarks on Ligations; Re-amputation at the Hip Joint for Osteo-Myleitis; Hæmorrhage on 6th day; Ligation of Primitive Iliac, by John H. Packard, Philadelphia. The common femoral was tied at the time of the disarticulation.

Dr. Jerfield, Boston, spoke in favor of learning to tie arteries "with the eyes toward heaven."

Dr. Post thought this very hazardous.

Dr. Connor, Cincinnati, reported a case where he had amputated in the upper one-third of thigh, found bone diseased, split down, nucleated the bone and left a good stump. Man recovered nicely. Thought by the use of Furneaux Jordan's method of amputation, the Esmarch bandage could be used, and there was no use for it in ligature of iliac.

IV. An anomalous case of Traumatic Aneurism. By Dr. J. Richardson, New Orleans. Bullet wound shot on inner side of leg; ball passed upward and out. Six months later, noticed enlargement between cicatrices. Employed compression, then ligated femoral under carbolic acid spray. Two years later, found circulation stopped in foot and leg, and mummification setting in. Amputated above middle of thigh, followed by gangrene. The photographs of the aneurism were shown. Writer dwells on "failure of leg to effect coagulation." No communication of aneurism with artery below. Dark matter of blood. Expressed the opinion that it was an arterio-venous aneurism. Read an additional paper on "Trephine in connection with Emphysema."

Dr. J. Ewing Mears, Philadelphia, spoke of his manner of passing drainage tubes through thoracic abscesses by means of hernia needles.

Dr. Campbell, Augusta, Ga., thought the trephine a very awkward instrument to use on ribs: and recommended the chain saw.

Dr. Richardson said: Had recommended trephine only in traumatic emphysema with fistula. Trephine, a very neat instrument and operation made in less than a minute. The chain saw jogged too much. Trephine should be about large enough to take up all the rib.

Adjourned to the amphitheater of the Medical College of Ohio, where Dr. Packard showed his method of ligating the primitive iliac, also his method of colotomy.

Association went into executive session for the election of new Fellows.

V. "On the Value of Early and Late Operations in Surgery." By S. D. Gross, Philadelphia. Spoke of the value of early operations in tumors and malignant growths. Importance of surgical interference in morbid growths. He said that diagnosis was a high art, and the majority of the profession were not sufciently good in it. He thought the word charlatan was not used widely enough in its sense. There are many knaves who treat malignant growths. In carcinoma, the knife cannot be used too early. All benign growths of rapid growths cannot be excised too soon. In case of repeated operation on carcinoma of the breast and its return, the knife should sweep away all the involved tissue; and the wound be treated on general principles.

Dr. Port spoke of the importance of removing growths not malignant. He recommended the removal of all such growths where not contra-indicated.

Dr. Moore said he really had nothing to say. There was nothing additional to say.

Dr. Gregory, of St. Louis: In tumors the change of cells was greater in the early part of the development than in the later. Irritation does not originate tumors. I am as successful in going into the peritoneum, as those I read of. Delay operations when the peritoneum is involved, otherwise operete as early as possible.

Dr. Gross did not speak further.

VI. Dislocation of the Astragalus. By Basil Morris, Washington. Spoke of the rarity of the case. Gave history of its occurrence in his own person and read answers from surgeons in regard to their experience and cases. Gave the authorities as advising; some reduction, some exsection, others attempts at reduction, then if failure excision. In his case, reduction was effected. He recommended gentleness in manipulation for reduction.

Drs. Moore, Werst, Dawson and Gunn had seen cases which they reported.

VII. "Excision of the Tarsus." By Dr. P. S. Connor, Cincinnati. He compared excision favorably with amputation. Claimed that it would tend to eradicate the disease and that the patient after recovery would have a very serviceable foot. The subject was treated with spirit and thoroughness, and the Fellows declined to discuss a subject already so admirably handled.

VIII. Trephining the Sternum for Removing Foreign Bodies from the Anterior Mediastinum. By S. Marks, Milwaukee, Wis. Related a case of gun-shot injury received during the late war. Ball remained behind the sternum until '70, when it was first discovered and removed by trephining. The pulsation of the pericardium could be plainly seen at the end of the operation. An interesting point was, that it showed how long foreign bodies could be borne near the heart.

IX. "Some questions with reference to Intracapsular Fracture of the Femur." By E. M. Moore, Rochester, N. Y. This elaborate paper was the result of extended observations on the human subject, a large number of specimens being produced by the reader.

X. "Fractures of the Neck of the Femur, with special reference to bony union of the Intra-Capsular Fracture." By N. Senn, Milwaukee, Wis. This gentleman produced a paper prodigious as to its amount of patient work and scholarship. It was replete with experiments on lower animals and decidedly "German" in its character. This and the preceding paper were discussed together, and, while all praised the skill and determined energy of Dr. Senn, yet they gave the preference rather to the paper of Dr. Moore, so full of practicability. Each paper after its kind, was certainly difficult to excel.

Dr. Moore said he had seen a case where a vascular frenum extended upon the head of the femur sufficient to give it enough nutrition to cause bony union, but he thought the case of Dr.

Senn's, reported to be a case of bony union, was not surgically a fracture within the capsule.

Dr. Campbell thought that the neck of the femur would not unite if it was held together. Thought we had better retain the old treatment of these "old broken hips."

Drs. Moore and Senn answered their critics briefly.

XI. "Nephrectomy for Carcinoma and partial Cholecystectomy for Gall Stone in the same subject." By S. W. Gross. A report of a case in brief and pithy style.

XII. "Operation for Permanent Closure of the Jaws. By J. Ewing Mears, Phila. Suggested excision of portion of ramus, condyloid and coronoid processes.

Dr. McGuire reported a similar case.

Dr. Port spoke of mechanical motion when there was any degree of extension. When there was no movement at all, then incision must be made.

XIII. "Removal of Meckel's Ganglion for relief of Trifacial Neuralgia with report of cases." By A. Vanderveer, Albany, N. Y. Reported three cases in which the operation was done; and wrote in its favor.

XIV. Dr. Campbell spoke in favor of the removal of the superior dental nerve.

Dr. Connor, in favor of resection of the infraorbital nerve.

Dr. Dawson reported a case of a young man, where he had removed nerves a half a dozen different times, each time followed by relief of pain for a season, then recurrence in a different quarter.

Dr. Vanderveer said that he had so far failed to find a reason why Meckel's ganglion should be removed, but the authorities recommended it, and he thought no one had ever had better success than that had in one of his cases.

XV. "Splint for extension of Wrist Joint." By J. M. Barton, Phila. Exhibited and explained.

XVI. "A Rectal Obturator." The instrument was exhibited and a paper read on the subject.

The Committee on Nominations, after expressing their deep regret at the positive refusal of Prof. Gross to act longer as president, reported the following names for election to the respective offices:

President, Dr. E. M. Moore, N. Y.

First Vice President, Dr. W. W. Dawson, Cincinnati.

Second Vice President, Dr. C. H. Maston, Alabama.

Secretary, Dr. J. R. Weist, Richmond, Ind.

Treasurer, Dr. J. H. Packard, Philadelphia.

Recorder, Dr. J. Ewing Mears, Philadelphia.

Council, Dr. P. S. Connor, Cincinnati.

Recommended the next meeting to be held at Washington, D. C., on the Wednesday preceding the meeting of the American Medical Association.

Report adopted.

A motion to have the applications for fellowship made directly to the Association, not through Council, lost.

On motion a vote of thanks was tendered the Reception Committee of Cincinnati.

On motion it was resolved that the Association have a banquet at each annual meeting at its own expense.

Drs. N. P. Dandridge, Cincinnati, Christ. Fenger, Chicago, W. E. Taylor, California, W. F. Peck, Davenport, Iowa, J. W. McCairn, Pittsburgh, and Y. M. Markoe, N. Y. were declared elected Fellows of the Association.

Dr. Kinlon moved that the names of the gentlemen signed to a paper criticizing the Code of Ethics of the American Medical Association, be referred to Council with instructions to report before the adjournment of this meeting. Carried.

Council reported as follows:

*Resolved:* That the Secretary be instructed to address to each of the members alleged to have criticized the Code of Ethics of the American Medical Association, requesting them, if the allegation be true, to resign. Carried unanimously.

A motion to increase the limit of the number of Fellows from 100 to 150, was lost.

A resolution of respect and esteem for the retiring president was offered by Dr. Dawson. Carried.

The President made the following closing remarks: I congratulate you on the success of the 4th annual meeting of the

American Surgical Association. I have tried to do all with impartiality. Now, as I am about to retire, I am sure the Association will be safe in the hands of my successor. I have no greater ambition than to hold your affection and esteem. I believe we have formed a successful institution and grounded on the right principle, and when I die, I hope that you will carry it on well. God bless you. Remain faithful to the interests of your profession to the end.

Adjourned, June 2d, 6 p. m.

Tne American Surgical Association is a noble-looking body of men. One can not look at its assembly and not see men of intellect. They are conservative surgeons. Not 50 per cent. of the applicants for fellowship were successful and a motion to increase the number was lost unanimously. The first 100 is not yet filled.

At a banquet given on the 2d day of the session, at the Gibson House, there were not a half dozen who were not members. A mentionable feature was the respect paid by the association to its president. Your correspondent has never seen its equal. The papers were so long and numerous as to preclude the possibility of much discussion, otherwise the 4th annual session of the American Association was regarded an eminent success.

E. S. McKEE, M. D.

June 2, 1883. Cincinnati.

